# GOLPH3 is a potential therapeutic target and a prognostic indicatior of poor survival in bladder cancer treated by cystectomy

**DOI:** 10.18632/oncotarget.4867

**Published:** 2015-08-22

**Authors:** Qing Zhang, Junlong Zhuang, Yongming Deng, Xiaozhi Zhao, Bo Tang, Dongwei Yao, Wei Zhao, Cunjie Chang, Qun Lu, Wei Chen, Shiwei Zhang, Changwei Ji, Lin Cao, Hongqian Guo

**Affiliations:** ^1^ Department of Urology, Drum Tower Hospital, Medical School of Nanjing University, Institute of Urology, Nanjing University, Nanjing 210008, Jiangsu, PR China; ^2^ College of Food Science and Technology, Nanjing Agricultural University, Nanjing 210095, Jiangsu, PR China; ^3^ Vazyme Biotech Co., Ltd, Nanjing 210000, Jiangsu, PR China; ^4^ MOE Key Laboratory of Model Animal for Disease Study, Model Animal Research Center, Nanjing Biomedical Research Institute, Nanjing University, Nanjing, Jiangsu 210061, China

**Keywords:** bladder cancer, GOLPH3, AKT/mTOR signalling, prognosis, survival

## Abstract

Golgi phosphoprotein 3 (GOLPH3) has been reported to be involved in the development of several human cancers. However, its clinical significance and biological role in bladder cancer remains unclear. In this study, we sought to analyze the GOLPH3 expression in bladder cancer samples and cells, and explore its clinical significance and biological role. We found that GOLPH3 was significantly increased in bladder cancer tissues and cells. Overexpression of GOLPH3 had significant correlation with poorer survival for bladder cancer patients treated by cystectomy. Knockdown of GOLPH3 inhibited the proliferation, migration and invasion of cancer cells, and tumor growth in a xenograft mouse model. GOLPH3 silencing inhibited AKT/m-TOR signaling, increased the cyclin-dependent kinase (CDK) inhibitor p27 and decreased the CDK regulator cyclin D1 and matrix metallopeptidase 9 (MMP9). Thus, GOLPH3 is likely to play important roles in bladder cancer progression via modulating AKT/mTOR signaling, and it is a novel prognostic biomarker and promising therapeutic target for bladder cancer.

## INTRODUCTION

Bladder cancer, primarily urothelial cell carcinoma, is the second most common genitourinary malignancy leading to significant morbidity and mortality [1]. Despite considerable progress in bladder cancer treatment, the prognosis of patients with locally advanced or muscle-invasive bladder cancer remains poor [2]. Although many molecular markers have been studied for their potential use in assessing the prognosis of bladder cancer, few molecular biomarkers have been shown to predict outcome [3, 4]. Moreover, novel alternative molecular therapeutic targets for bladder cancer need to be discovered. Therefore, it is important to identify reliable molecular prognostic markers and therapeutic targets in clinical practice for the treatment of bladder cancer.

Golgi phosphoprotein 3 (GOLPH3), also known as GPP34, is a newly identified membrane protein in the trans-Golgi matrix, which plays a role in anterograde and retrograde Golgi trafficking [5–7]. Recently, GOLPH3 has been shown to be involved in tumorigenesis. GOLPH3 localizes at human chromosome 5p13, a region that is frequently amplified in multiple solid tumors [8, 9]. It has been reported that overexpression of GOLPH3 promotes cell transformation by enhancing the activity of the serine/threonine kinase mTOR in breast cancer [10, 11]. However, the clinical significance and biological role of GOLPH3 in bladder cancer remains unclear.

Here, we show that GOLPH3 is frequently overexpressed in bladder cancer treated by cystectomy, and this highexpression is significantly associated with worse prognosis. GOLPH3 silencing in bladder cancer cells decrease the cell proliferation, migration and invasion likely by inhibiting AKT/mTOR signaling. *In vivo* studies further demonstrate that GOLPH3 silencing dramatically inhibits xenograft tumor growth and may be a promising therapeutic target. Together, our data highlight an important role for GOLPH3 in controlling bladder cancer progression and its promise as a therapeutic target and novel prognostic indicator for poor survival in bladder cancer.

## RESULTS

### GOLPH3 protein and mRNA expression in bladder cancer tissues and cell lines

**Figure 1:**
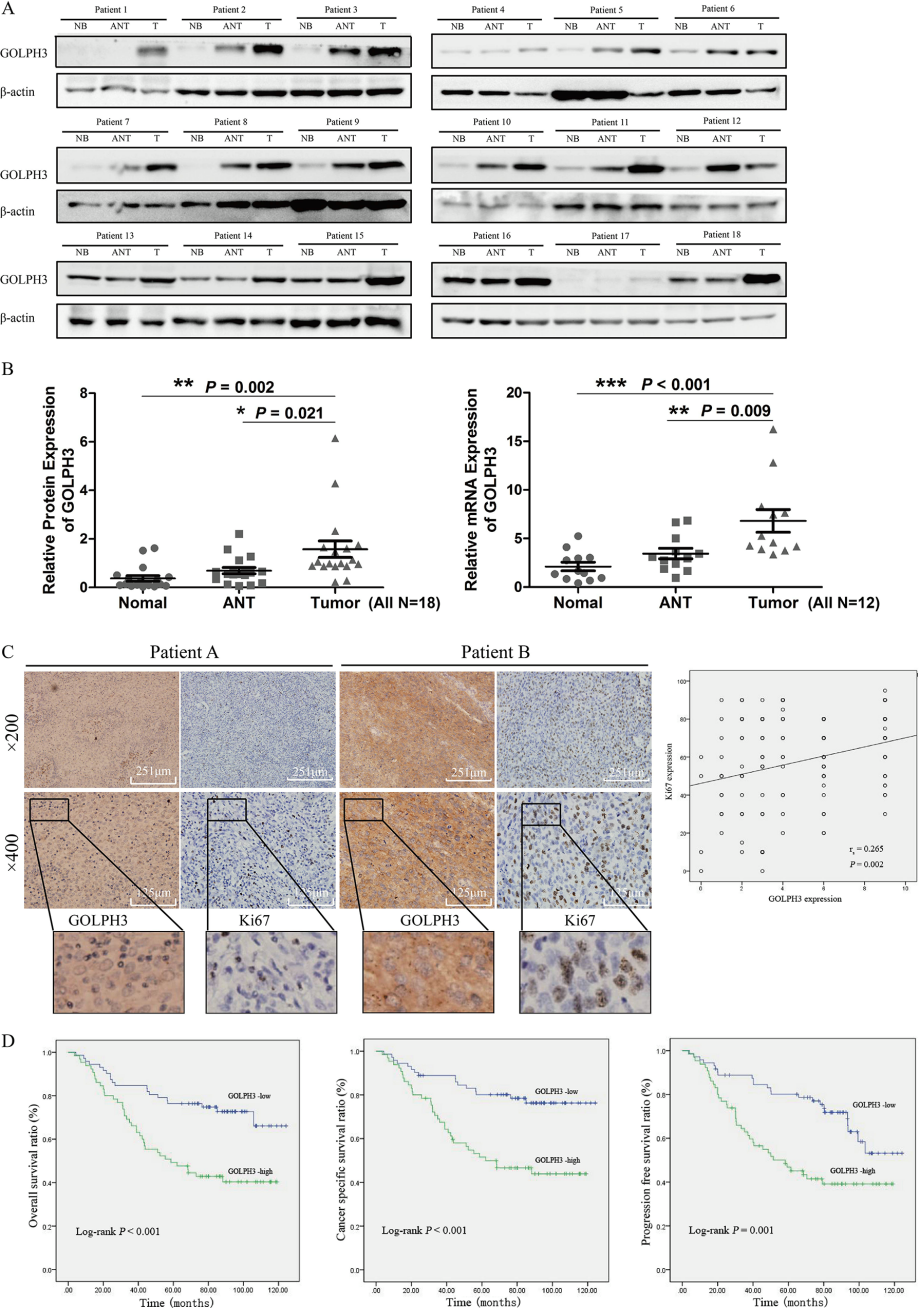
GOLPH3 expression is increased in human bladder cancer tissues and is related to the expression of Ki67 and the prognosis of patients Western blot (18 paired) **A.** and real-time PCR (12 paired) **B.** analysis of GOLPH3 expression in paired primary bladder cancer tissues (T), adjacent noncancerous tissues (ANT) and Normal bladder tissues (NB). IHC staining analysis of the association of GOLPH3 and Ki67 protein expression in paraffin-embedded, archived bladder cancer tissues **C.** Kaplan-Meier survival analysis of overall survival, cancer-specific survival and progression-free survival **D.** for all 137 bladder cancer patients with low GOLPH3-expressing (*n* = 72) versus high GOLPH3-expressing tumors (*n* = 65). The log-rank test was used to calculate *P*-values.

**Figure 2:**
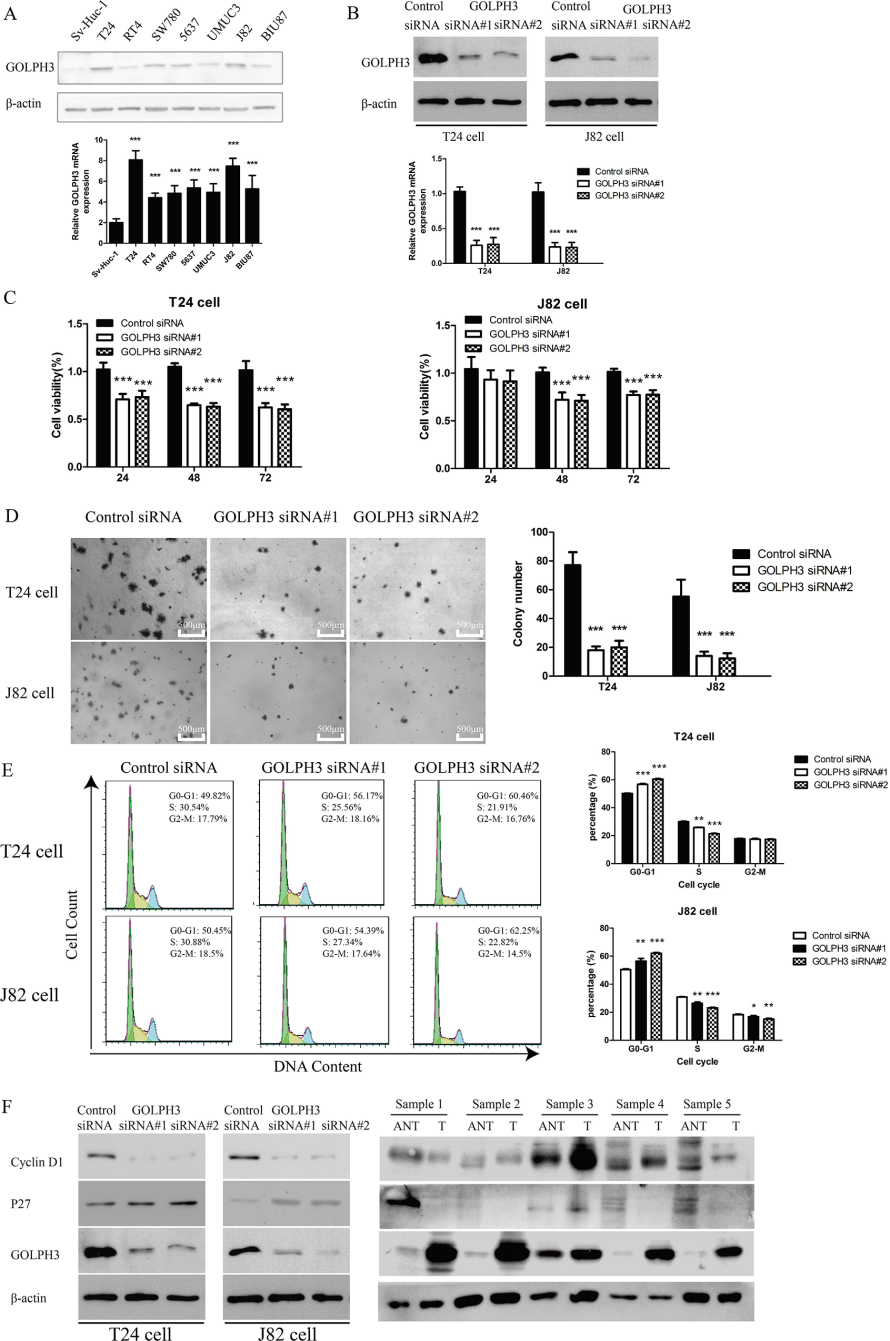
GOLPH3 silencing inhibited the proliferation and tumorigenicity of bladder cancer cells *in vitro* Western blot and real-time PCR analysis **A.** of GOLPH3 expression in the immortalized normal human uroepithelium cell line SV-HUC-1, and 7 other bladder cancer cells. GOLPH3 expression of negative control siRNA-transfected and GOLPH3-silenced T24 and J82 cells was also analyzed by western blot and real-time PCR **B.** analysis. CCK8 assays **C.** indicated that the growth rates decreased in GOLPH3-silenced cells. Inhibition of T24 and J82 cells anchorage independent colony formation capacity by GOLPH3 siRNA **D.** Flow cytometric analysis **E.** indicated that GOLPH3 stimulates the G1-S phase transition of bladder cancer cells, and representative micrographs (left) and quantification (right) are shown. Western blot analysis **F.** of cell-cycle regulators (Cyclin D1 and P27) in GOLPH3 siRNA-infected cells and 5 paired primary bladder cancer tissues (T) and adjacent noncancerous tissues (ANT).

Western blot and real-time PCR analyses showed that the GOLPH3 expression level in most human bladder cancer tissues was higher than that in paired ANT and NB (Figure 1A and 1B). Western blot and real-time PCR analysis showed that 17 of 18 (94.4%) patients had a high GOLPH3 protein expression level and 11 of 12 (91.7%) patients had a high GOLPH3 mRNA expression level compared with paired ANT and NB samples. Furthermore, comparative analysis revealed that the GOLPH3 protein and mRNA expression were significantly increased in all of the 7 tested bladder cancer cell lines compared with the normal human uroepithelium cell SV-HUC-1 (Figure 2A). These results suggest that GOLPH3 expression is increased in most human bladder cancer.

### Immunohistochemical analysis of GOLPH3 expression in archived bladder cancer samples and its relationship to clinicopathological parameters

To investigate the relationship between GOLPH3 expression and the clinicopathologic characteristics of bladder cancer, GOLPH3 and Ki67 expression was examined in 137 paraffin-embedded, archived bladder cancer tissues by IHC staining. The statistical analysis of the IHC staining is summarized in Table 1. Statistical analysis revealed that the GOLPH3 levels are not associated with the grade, T classification, N classification, or multiplicity classification (Table 1; all *P* > 0.05), but they are associated with age (≤ 65 y vs. > 65 y; *P* < 0.05), which may means that age may effects the function of GOLPH3 gene. Further statistical analysis revealed that the GOLPH3 levels are only associated with the T classification (NMI or MI) in older people (> 65 yr) (Table 2; *P* = 0.047). Strikingly, we found that the areas in the bladder cancer specimens displaying high levels of GOLPH3 staining also had strong Ki67 staining signals, and areas with low GOLPH3 expression had weakly detectable Ki67 expression (Figure 1C). Statistical analysis indicated that the GOLPH3 expression was strongly associated with the Ki67 expression level (*P* = 0.002; Figure 1C, Table 3), suggesting that GOLPH3 overexpression may contribute to the proliferation of human bladder cancer.

**Table 1:** Association between GOLPH3 expression and the clinicopathologic characteristics of 137 patients treated with radical cystectomy for urothelial carcinoma of the bladder

Characteristic	Total cases	GOLPH3 expression		*P* value
		Low	High	
Total	137	72	65	
Gender				0.783
Female	24(17.5%)	12(16.7%)	12(18.5%)	
Male	113(82.5%)	60(83.3%)	53(81.5%)	
Age				0.031
≤ 65 y	66(48.2%)	41(56.9%)	25(38.5%)	
< 65 y	71(51.8%)	31(43.1%)	40(61.5%)	
Grade				0.067
G1–G2	42(30.7%)	27(37.5%)	15(23.1%)	
G3	95(69.3%)	45(62.5%)	50(76.9%)	
pT status				0.145
NMI	68(49.6%)	40(55.6%)	28(43.1%)	
MI	69(50.4%)	32(44.4%)	37(56.9%)	
pN status				0.315
N0	120(87.6%)	65(90.3%)	55(84.6%)	
N1–N2	17(12.4%)	7(9.7%)	10(15.4%)	
Multiplicity				0.877
Unifocal	102(74.5%)	54(75.0%)	48(73.8%)	
Multifocal	35(25.5%)	18(25.0%)	17(26.2%)	

NMI = Non-muscle invasive bladder cancer; MI = Muscle invasive bladder cancer.

**Table 2:** Association between GOLPH3 expression and the clinicopathologic characteristics in older patients (>65 yr) with bladder cancer

Characteristic	Total cases	GOLPH3 expression		*P* value
		Low	High	
Total (>65 yr)	71	31	40	
Gender				0.300
Female	13	4(12.9%)	9(22.5%)	
Male	58	27(87.1%)	31(77.5%)	
Grade				0.747
G1-G2	15	6(19.4%)	9(22.5%)	
G3	56	25(80.6%)	31(77.5%)	
pT status				0.047
NMI	34	19(61.3%)	15(37.5%)	
MI	37	12(38.7%)	25(62.5%)	
pN status				0.722
N0	62	28(90.3%)	34(85.0%)	
N1-N2	9	3(9.7%)	6(15.0%)	
Multiplicity				0.425
Unifocal	54	25(80.6%)	29(72.5%)	
Multifocal	17	6(19.4%)	11(27.5%)	

NMI = Non-muscle invasive bladder cancer; MI = Muscle invasive bladder cancer.

**Table 3:** (a) Univariate and (b) multivariate analyses for the effects of GOLPH3 expression on the progression-free, cancer-specific and overall survival of 137 patients treated with radical cystectomy for urothelial carcinoma of the bladder

Variable	Progression Free			Cancer Specific			Overall Survival		
	HR	95%CI	*P* value	HR	95%CI	*P* value	HR	95%CI	*P* value
(a)									
Gender (Male vs Female)	0.638	0.345–1.180	0.152	0.585	0.306–1.119	0.105	0.510	0.283–0.918	0.025
Age (≤65 y vs >65 y)	1.310	0.793–2.163	0.292	1.598	0.910–2.807	0.103	1.690	0.994–2.876	0.053
Grade(G1–G2 vs G3)	2.272	1.218–5.235	0.010	2.093	1.047–4.183	0.037	2.231	1.154–4.314	0.017
pT status (NMI vs MI)	4.223	2.385–7.477	< 0.001	4.197	2.230–7.900	< 0.001	3.469	1.966–6.121	< 0.001
pN status (N0 vs N1–N2)	6.077	3.306–11.173	< 0.001	6.206	3.282–11.736	< 0.001	5.715	3.115–10.483	< 0.001
Multiplicity (Unifocal vs Multifocal)	1.790	1.056–3.035	0.031	1.343	0.735–2.454	0.338	1.465	0.840–2.557	0.179
GOLPH3 expression (Low vs High)	2.406	1.437–4.027	0.001	3.041	1.680–5.506	< 0.001	2.631	1.528–4.529	< 0.001
Ki67 expression (Low vs High)	3.379	1.924–5.934	< 0.001	4.118	2.110–8.040	< 0.001	3.294	1.827–5.940	< 0.001
**Variable**	**Progression Free**			**Cancer Specfic**			**Overall Survival**		
	**HR**	**95%CI**	***P* value**	**HR**	**95%CI**	***P* value**	**HR**	**95%CI**	***P* value**
(b)									
Gender (Male vs Female)	0.644	0.338–1.229	0.183	0.569	0.286–1.131	0.108	0.484	0.259–0.904	0.023
Age (≤65 y vs > 65 y)	1.111	0.641–1.927	0.707	1.274	0.684–2.374	0.445	1.436	0.797–2.587	0.228
Grade(G1-G2 vs G3)	1.431	0.735–2.787	0.292	1.159	0.553–2.427	0.696	1.351	0.671–2.720	0.400
pT status (NMI vs MI)	2.558	1.370–4.777	0.003	2.424	1.212–4.848	0.012	1.997	1.065–3.745	0.031
pN status (N0 vs N1–N2)	3.069	1.543–6.107	0.001	3.364	1.625–6.963	0.001	3.499	1.731–7.071	< 0.001
Multiplicity (Unifocal vs Multifocal)	1.370	0.786–2.386	0.267	1.012	0.538–1.904	0.971	1.192	1.122–3.392	0.557
GOLPH3 expression (Low vs High)	1.920	1.130–3.260	0.016	2.257	1.235–4.126	0.008	1.951	1.122–3.392	0.018
Ki67 expression (Low vs High)	2.337	1.282–4.259	0.006	2.515	1.239–5.107	0.011	1.951	1.041–3.655	0.037

HR = hazard ratio; CI = confidence interval; NMI = Non-muscle invasive bladder cancer; MI = Muscle invasive bladder cancer.

### Overexpression of GOLPH3 correlates with poor prognosis in human bladder cancer

To investigate the relationship between GOLPH3 expression and survival for patients with bladder cancer, GOLPH3 expression in 137 paraffin-embedded, archived bladder cancer tissues was studied by IHC staining, and follow up data were analyzed. Kaplan-Meier survival curves and log-rank test survival analyses showed that the overall survival, cancer-specific survival and progression-free survival of patients with low levels of GOLPH3 were significantly better than that for patients with high levels of GOLPH3 (all *P* < 0.05; Figure 1D). Univariate and multivariate analyses indicated that clinical grade stage, T classification, N classification, and the expression of GOLPH3 and Ki67 were prognostic factors (Table 3), suggesting that GOLPH3 may be an independent prognostic factor for the survival of patients with bladder cancer treated by cystectomy.

### Effects of GOLPH3 silencing on cell proliferation and tumorigenicity *in vitro*

To further examine the functional role of GOLPH3 in bladder cancer cells, we specifically knocked down its expression using siRNA in T24 and J82 cells, which express the highest levels of endogenous GOLPH3 of 7 tested bladder cancer cell lines. We observed that the GOLPH3 protein and mRNA levels were significantly reduced in cells transfected with GOLPH3-specific siRNA compared with cells transfected with negative control siRNA (Figure 2B). We next studied the impact of GOLPH3 silencing on cell proliferation *in vitro*. The results of a CCK8 assay showed that GOLPH3 silencing significantly reduced the cell viability in both cell lines tested compared with control cells (Figure 2C). An anchorage-independent growth assay also revealed that GOLPH3 silencing in T24 and J82 cells resulted in a reduced colony formation ability (All *P* < 0.001; Figure 2D).

To further investigate the mechanism of GOLPH3-mediated proliferation, flow cytometry assays were performed. As shown in Figure 2E, silencing GOLPH3 in T24 and J82 cells increased the percentage of cells in the G1-G0 phase and decreased the percentage of S phase cells. These results suggest that GOLPH3 contributes to the G1-S transition in bladder cancer cells. Importantly, we found that the p27 expression level was drastically increased in GOLPH3-silenced cells accompanied by significantly reduced levels of cyclin D1 compared with control cells (Figure 2F). Consistently, the expression of p27 was significantly decreased, whereas cyclin D1 was increased in bladder cancer tissues compared with matched ANT (Figure 2F).

### Effects of GOLPH3 silencing on cell migration and invasion *in vitro*

**Figure 3:**
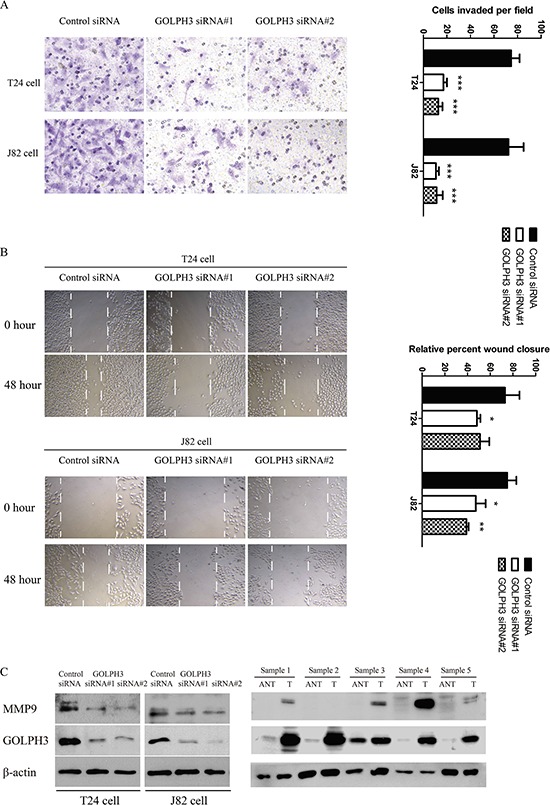
GOLPH3 inhibition attenuated the invasion and migration capability of T24 and J82 cells *in vitro* **A.** Matrigel invasion assays showed that GOLPH3 siRNA-transfected cells resulted in low penetration through Matrigel-coated membranes compared with control cells. **B.** A scratch migration assay demonstrated that GOLPH3 decreased tumor cell migration. Western blot analysis **C.** of the metastasis associated protein MMP9 in GOLPH3 siRNA-infected cells and 5 paired primary bladder cancer tissues (T) and adjacent noncancerous tissues (ANT).

In a matrigel invasion assay, knockdown of GOLPH3 significantly suppressed the invasiveness of T24 and J82 cells (Figure 3A). The average cell counts crossing the matrigel-coated membrane in one high power field were 74.67 ± 7.09, 17.00 ± 3.00 and 12.67 ± 3.51 for control, GOLPH3 siRNA#1, and siRNA#2, respectively, in T24 cells (*P* < 0.001) and 72.33 ± 12.90, 10.67 ± 2.52 and 11.33 ± 5.13 for control, GOLPH3 siRNA#1 and siRNA#2, respectively, in J82 cells (*P* < 0.001). Moreover, scratch migration assays showed that GOLPH3 silencing significantly suppress the migration capability of both cancer cell lines (Figure 3B). Furthermore, the MMP9 expression level was drastically reduced in GOLPH3-silenced cells compared with control cells (Figure 3C) accompanied by significantly increased levels of MMP9 in bladder cancer tissues compared with ANT (Figure 3C).

### GOLPH3 may activates AKT/mTOR signaling to promote the progression of human bladder cancer

**Figure 4:**
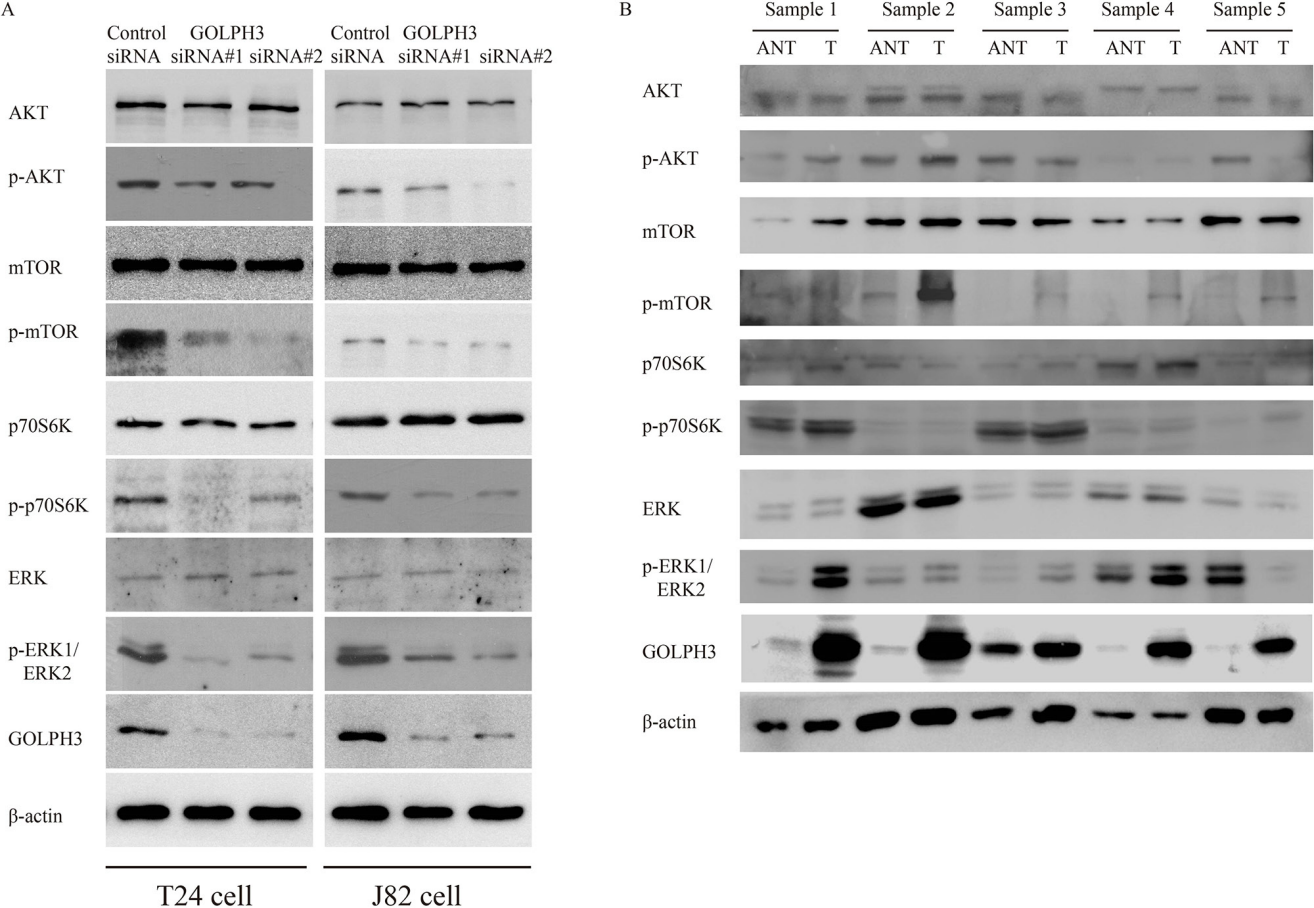
The effects of GOLPH3 inhibition on AKT/mTOR signaling Western blot analysis of AKT/mTOR signaling (p-AKT, total AKT, p-mTOR, total mTOR, p-p70S6K, total p70S6K), p-ERK1/ERK2, and total ERK in GOLPH3 siRNA-infected cells **A.** and 5 paired primary bladder cancer tissues (T) and adjacent noncancerous tissues (ANT) **B.**

It has been reported that overexpression of GOLPH3 promotes cell transformation by enhancing the activity of the serine/threonine kinase mTOR in breast cancer [11, 16]. To test the hypothesis that GOLPH3 also activates AKT/mTOR signaling in bladder cancer, western blotting was performed to examine the protein level of AKT/mTOR signaling pathway components. Inhibition of GOLPH3 by siRNA resulted in decreased expression of p-AKT, p-mTOR, p-p70S6K and p-ERK1/ERK2 in T24 and J82 cells; however, the protein levels of AKT, mTOR, p70S6K and ERK1/ERK2 in the two tested cell lines were not significantly different (Figure 4A). As shown in Figure 4B, the expression of p-AKT, p-mTOR, p-p70S6K and p-ERK1/ERK2 was consistently significantly increased, whereas AKT, mTOR, p70S6K and ERK1/ERK2 were not significantly different in human bladder cancer tissues and matched ANT. These results indicate that GOLPH3 may activate AKT/mTOR signaling to promote the progression of human bladder cancer.

### GOLPH3 silencing inhibits the tumorigenicity of bladder cancer *in vivo*

**Figure 5:**
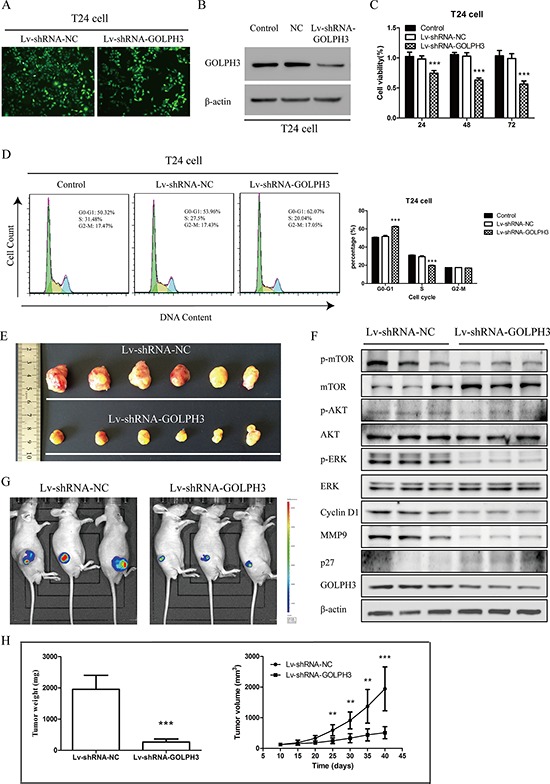
GOLPH3 silencing inhibits the tumorigenicity of bladder cancer cells *in vitro* and *in vivo* **A.** The establishment of stable T24 bladder cancer cell lines expressing GOLPH3 and negative control shRNAs. The GFP expression ratio of T24 cells was greater than 90% 24 h post-cotransfection. **B.** GOLPH3 expression in stable T24 cells expressing GOLPH3 and negative control shRNAs was analyzed by western blot analysis. CCK8 assays **C.** indicated that the growth rates decreased in GOLPH3-silenced T24 cells. Flow cytometric analysis **D.** indicated that GOLPH3 stimulates the G1-S-phase transition of bladder cancer cells, and representative micrographs (left) and quantification (right) are shown. Stable knockdown of GOLPH3 by Lv-shRNA-GOLPH3 **F.** inhibits the growth of T24-derived xenografts in nude mice **E.** and **G.** Macrographic images show that the tumor size of the Lv-shRNA-GOLPH3 group was markedly smaller on the 40th day than those of the Lv-shRNA-NC group. The growth curves of the tumor xenografts and the final tumor weights of the Lv-shRNA-GOLPH3 group was decreased compared with those of the Lv-shRNA-NC group **H.** The tumor inhibition effect induced by GOLPH3 silencing *in vivo* correlate with the inhibition of AKT/mTOR signaling and Cyclin D1, MMP9 and increased p27 expression (F).

To investigate whether GOLPH3 silencing had inhibitory effects on tumor growth *in vivo*, we first established stable T24 bladder cancer cell lines expressing GOLPH3 or negative control short hairpin RNAs (Figure 5A and 5B). The results of CCK8 assays demonstrated that inhibition of GOLPH3 significantly reduced cell viability (Figure 5C) by blocking the G1-S-phase transition in stable T24 cells expressing GOLPH3 short-hairpin RNAs (Figure 5D). Then, a nude mouse xenograft model of stable T24 bladder cancer cell lines expressing GOLPH3 and negative control short-hairpin RNAs was established (Figure 5E, and 5G). As shown in Figure 5H the tumor volume of Lv-shRNA-GOLPH3 mice from day 25 to the end of the experiment was significantly smaller than that of Lv-shRNA-NC mice (all *P* < 0.01). Knockdown GOLPH3 expression resulted in a significant decrease in the tumor volume and weight as measured at the end of the experiment at day 40 when compared with Lv-shRNA-NC mice (*P* < 0.001; Figure 5E, and 5H). Moreover, inhibition of GOLPH3 consistently suppressed expression of p-AKT, p-mTOR, p-ERK1/ERK2, Cyclin D1, MMP9 and increased of p27 in T24 xenograft tumors; however, the protein levels of AKT, mTOR and ERK1/ERK2 were not significantly different (Figure 5F). These results further indicate that GOLPH3 connected with AKT/mTOR signaling, Cyclin D1, MMP9 and p27 in bladder cancer.

### GOLPH3 is a promising therapeutic target for bladder cancer

**Figure 6:**
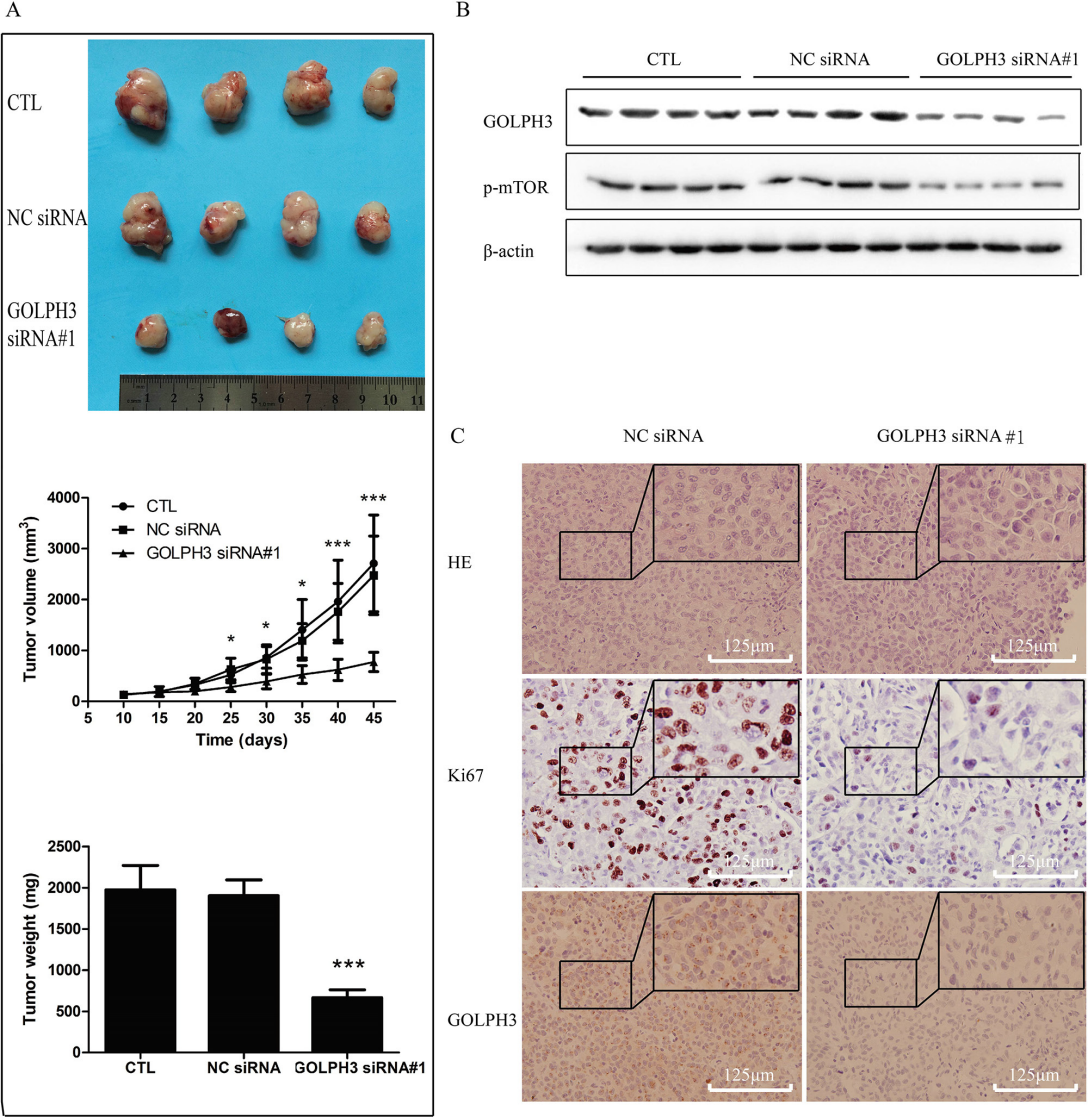
GOLPH3 may as a promising therapeutic target for bladder cancer *in vivo* **A.** Knockdown of GOLPH3 by the intratumoral injection of siRNA inhibits the growth of T24-derived xenografts in nude mice. Top, macrographic image showing that the tumor size in the GOLPH3 siRNA group was markedly smaller on the 45th day after tumor cells inoculation than that of the groups injected with PBS and control siRNA; middle, growth curves of the tumor xenografts; bottom, the final tumor weights of the GOLPH3 siRNA group were decreased compared with that of the PBS and negative control siRNA groups. Intratumoral injection of GOLPH3 siRNA inhibited the protein expression of GOLPH3, p-mTOR and Ki67 as determined by western blotting **B.** and IHC **C.** analysis in T24 cell derived xenografts.

To further examine whether GOLPH3 had potential as the therapeutic target in human bladder cancer, a nude mouse xenograft model of T24 cells was established, and intratumoral injection with PBS, negative control siRNA and the GOLPH3 siRNA#1 complex in nude mice with T24 cells tumor burden was performed. As shown in Figure 6A, the tumor volume in the GOLPH3 siRNA#1 group starting from day 25 to the end of the experiment was significantly smaller than in the PBS and control siRNA groups (*P* < 0.05). Furthermore, GOLPH3 siRNA#1 resulted in a significant decrease in tumor volume and weight as measured at the end of the experiment at day 45 compared with control siRNA (*P* < 0.001; Figure 6A). In addition, the relative protein expression level of GOLPH3, p-mTOR and Ki67 in GOLPH3 siRNA#1 tumors was significantly decreased compared with control siRNA tumors (Figure 6B and 6C).

## DISCUSSION

The key findings of this study are that GOLPH3 is overexpressed in most bladder cancer tissues and cell lines, which predicts poor survival for bladder cancer patients treated by cystectomy. Knockdown of GOLPH3 decreased the proliferation, migration and invasion of bladder cancer cells and the tumor growth in xenograft mice, which may be associated with GOLPH3 modulation of AKT/mTOR signaling. This study provides new insight and strong evidence that GOLPH3 is functionally important in the progression of bladder cancer, it may serve as novel prognostic indicator for poor survival and is a target for bladder cancer therapy.

Tumorigenesis is a complex multistep process characterized by uncontrolled cell growth and tumor formation, and it is largely associated with the progressive accumulation of various genetic and epigenetic alterations in genes and proteins that regulate cell proliferation [17, 18]. Therefore, the identification of genes and their products that lead to tumorigenesis is critical for providing new diagnostic and prognostic methods and potential therapeutic targets.

GOLPH3 has been implicated in protein trafficking, receptor recycling, and protein glycosylation and is a potential links between these cellular processes and tumorigenesis [19]. Previously, it has been reported that GOLPH3 is amplified at the 5p13 region, and high expression of GOLPH3 has been suggested to be a predictor of poor prognosis in several types of cancer, including breast cancer [11, 16], renal cell carcinoma [13], and esophageal squamous cell carcinoma [20, 21]. However, the clinical significance and biological role of GOLPH3 in bladder cancer remains unclear.

Here, we first found that GOLPH3 was significantly increased in a large cohort of human bladder cancer tissues (Figure 1A and 1B). However, the GOLPH3 levels were not significantly correlated with clinicopathological characteristics except for the age of the patients with bladder cancer in this cohort (Table 1), possibly due to the limited number of patients. Then, the further studies were conducted to explore the association between GOLPH3 expression levels and the clinicopathologic characteristics in older people (> 65 yr) (Table 2). We found that the GOLPH3 levels are associated with the T classification (NMI or MI) in older people (Table 2; *P* = 0.047), which indicates that increased GOLPH3 expression is associated with the progression of bladder cancer in older patients. Furthermore, we strikingly found that the expression of GOLPH3 was strongly associated with the Ki67 expression level (*P* = 0.002; Figure 1C), suggesting that GOLPH3 overexpression may contribute to the proliferation and tumorigenicity of bladder cancer. Moreover, survival analysis showed that patients with high GOLPH3 expression have worse overall cancer-specific and progression-free survival than patients with low levels of GOLPH3 (Figure 1D). Moreover, univariate and multivariate analyses further suggested that GOLPH3 is an independent predictor of the survival of patients treated by cystectomy and is associated with bladder cancer progression.

In this study, we found that GOLPH3 protein and mRNA expression are significantly increased in T24 and J82 bladder cancer cells compared with seven other bladder cancer cell lines and the immortalized normal human uroepithelium cell line SV-HUC-1 (Figure 2A). In an attempt to determine the potential of GOLPH3 as a therapeutic target, we used siRNA and shRNA to knockdown its expression and analyzed its phenotype and underlying mechanism. We found that GOLPH3 knockdown strikingly inhibited the proliferation and growth of bladder cancer cells *in vitro* and *in vivo* (Figures 2, 5 and 6). The result of flow cytometric assays revealed that inhibition of GOLPH3 induced G1-S-phase arrest. Furthermore, we demonstrated that the mechanism of GOLPH3-mediated proliferation was linked to alterations in the expression of the cell-cycle inhibitor p27 and the CDK regulator cyclin D1. Strikingly, these results from bladder cancer cells *in vitro* and *in vivo* are consistent with the protein expression level in human bladder cancer tissues compared with paired ANT (Figure 2F). These results are also consistent with the antitumor activity resulting from the inhibition of GOLPH3 expression in RCC [13] and breast cancer [11, 16]. Consequently, GOLPH3 appeared to be a critical factor for proliferation and tumorigenicity; therefore, it should be a good therapeutic target for halting the proliferation of bladder cancer.

In addition, our study demonstrates that knockdown of endogenous GOLPH3 in bladder cancer cells greatly reduces the cell migration and invasion capacity *in vitro* (Figure 3A and 3B), suggesting that GOLPH3 plays an important role in bladder cancer invasiveness. These results indicate that high expression of GOLPH3 may contribute to the malignancy (invasion and metastasis) of bladder cancer. The role of GOLPH3 in bladder cancer cell migration and invasion may associated with MMP9 expression, and its underlying mechanism needs to be explored further. Our study shows that the distinct association between GOLPH3 and tumor invasiveness may have therapeutic and prognostic implications.

Recently, mTOR, a key downstream effector of the phosphoinositide 3- kinase (PI3K)/AKT signaling pathway, has been recognized to play a crucial role in controlling cancer cell growth [22]. AKT and mTOR function as “master switch” proteins in cancer cells to modulate metabolism, cell cycle, and apoptosis [23]. Activated mTOR in turn activates downstream targets including p70S6 kinase-1 and initiation factor 4E-binding protein 1 (4E-BP1). Inhibition of AKT/mTOR signaling results in a wide variety of effects in normal and malignant cells, including the induction of apoptosis and inhibition of cell-cycle progression, cell growth, angiogenesis, endothelial cell proliferation, and protein translation [24, 25].

In this study, we evaluated the expression status of crucial biomarkers of the AKT/mTOR pathway, and the results revealed that p-mTOR, p-AKT, p-P70S6K transactivity was decreased in GOLPH3-silenced cells and xenograft tumors, suggesting that GOLPH3-silenced proliferation and tumorigenesis may be due to inhibition of AKT/mTOR signaling. The signaling inhibition could also be monitored by the levels of changes of established tumor markers such as extracellular signal regulated kinase (ERK1/2). These bladder cancer cell results *in vitro* and *in vivo* are consistent with the protein expression of human bladder cancer tissues compared with paired ANT.

To further explore whether GOLPH3 has the potential to act as a therapeutic target for bladder cancer, a nude mouse xenograft model of T24 cells was established, and intratumoral injection with GOLPH3 siRNA#1 was performed in the T24 cells established in the xenograft nude mouse model. It is surprising that intratumoral injection with GOLPH3 siRNA#1, which induced the inhibition of GOLPH3 expression, significantly inhibited the growth of bladder cancer cells in nude mice. This *in vivo* therapeutic effect resulting from intratumoral injection with GOLPH3 siRNA#1 was consistent with results showing that low GOLPH3 expression correlates with better survival than that of patients with high levels of GOLPH3. Strikingly, we found that the areas in T24 xenograft tumor specimens displaying high levels of GOLPH3 staining demonstrated strong Ki67 staining signals, and areas with low GOLPH3 expression had weakly detectable Ki67 expression (Figure 6C). These results are consistent with the results of human bladder cancer tissues, further suggesting that high GOLPH3 expression may contribute to the proliferation of bladder cancer and indicating that GOLPH3 may be a good therapeutic target for halting the proliferation and tumorigenesis of bladder cancer.

There are several limitations to our study. First, our study was a single hospital retrospective study. Future studies based on a multiple centers or a community-based prospective study with a more extensive collection of potential cofounders are also required. In addition, the case population in our study are only cytectomy specimens, which included mostly high grade urothelial carcinoma. Therefore, the observation may not be applicable to low grade tumors. Moreover, an *in vivo* metastasis assay and studies of the underlying mechanism of the observed migration and invasion should be performed to further validate the role of GOLPH3 in metastasis in human bladder cancer.

In conclusion, this study suggests that GOLPH3 is overexpressed in most bladder cancers treated by cystectomy and plays an important role in human bladder cancer progression by modulating AKT/mTOR signaling. Full understanding of the precise role of GOLPH3 in human bladder cancer may provide an opportunity for developing a novel therapeutic strategy by suppressing the expression of GOLPH3. In addition, GOLPH3 has potential as a relevant clinical indicator of disease progression and a prognostic marker for patient survival in human bladder cancer

## MATERIALS AND METHODS

### Patients information and tissue specimens

Between January 2004 and December 2008, 182 consecutive patients with bladder cancer who underwent a radical cystectomy with pelvic and iliac lymphadenectomy at Drum Tower Hospital, Medical School of Nanjing University, were included in this study. Eighteen patients who received preoperative neoadjuvant therapy, including either radiation or chemotherapy, 14 patients who were lost to follow-up, and 13 patients with another type of histology, including adenocarcinoma, squamous cell carcinoma, small-cell carcinoma, and sarcoma, were excluded from further analysis. No patients had distant metastases at the time of cystectomy. Human bladder cancer paraffin blocks of the remaining 137 patients were analyzed for IHC study. Tumor stage and grade were defined according to Unio Internationale Contra Cancrum and WHO classification as previously described [12]. The clinicopathologic characteristics of the patients are summarized in Table 1.

Bladder cancer tissue samples were obtained from 23 untreated patients who underwent a cystectomy for bladder cancer at the Department of Urology in our hospital between March 2012 and October 2014. All tumors were validated as urothelial carcinoma. Normal bladder (NBs) tissue samples were surgically excised from the same patients (5 cm distances from the tumor) and the adjacent noncancerous tissues (ANTs) were excised within a radius of 5 cm around the cancers tissue without cancer. Tissue samples were immediately snap frozen in liquid nitrogen. NBs and ANTs were both confirmed as normal by pathologic histological examination. For these samples, the tumor grade and stage were available; however, no follow-up data were available. The study protocol was approved by the Ethics Committee of Drum Tower Hospital, Medical School of Nanjing University (Nanjing, China), and written informed consent was obtained from each patient.

### Cell lines

The human bladder cancer cell lines T24 and J82 and the immortalized normal human uroepithelium cell line SV-HUC-1 were purchased from American Type Culture Collection (Rockville, MD, USA). An additional five human bladder cancer cell lines, RT4, 5637, UMUC3, SW780 and BIU87, were obtained from our institute. All of these cells were cultured in RPMI 1640 medium (HyClone Laboratories, Logan, UT, USA) with 10% fetal bovine serum (FBS). All cells were cultured in a sterile incubator maintained at 37°C with 5% CO_2_.

### Immunohistochemistry

Paraffin-embedded bladder cancer specimens were cut into 4 μm sections. Immunohistochemical staining was performed as described previously [13–15]. GOLPH3 and Ki67 expression was assessed by evaluating the proportion and intensity of positively stained carcinoma cells. A score was assigned to represent the estimated percentage of positively stained carcinoma cells as follows: 0: none, 1: ≤ 50%, 2: 50–75%, and 3: ≥ 75%. An intensity score was assigned to represent the average estimated intensity of staining in positive carcinoma cells as follows: 0: none, 1: weak, 2: intermediate, 3: strong. The proportion score and intensity score were multiplied to obtain a total score ranging from 0 to 9. The IHC results were classified based on total scores with 0 to 4 classified as low expression and 5 to 9 indicating high expression [14]. The IHC results were independently judged by two trained uropathologists. Differences in evaluations were discussed using a double headed microscope until a consensus was reached.

### Western blotting

Tissues or cells were lysed in lysis buffer containing protease inhibitor cocktail. Following quantification, the protein lysates were resolved in an SDS-PAGE gel, transferred to a PVDF membrane (Millipore, Billerica, MA, USA), and immunoblotted with an antibody directed against GOLPH3, cyclin D1, p27, MMP9, AKT, p-AKT, mTOR, ERK, S6K (Proteintech Inc., Chicago, IL,USA), p-mTOR, p-ERK, p-S6K(Cell Signaling Technology Inc., Danvers, MA, USA), or β-actin (Vazyme, Piscataway, NJ, USA) and then a horseradish peroxidase-conjugated secondary antibody (Vazyme, Piscataway, NJ, USA). Bound antibody was visualized using standard chemical luminescence methodology. β-actin was used as a loading control.

### Real-time quantitative PCR

Total RNA was isolated from tissue specimens or cells using the TRIzol reagent (Invitrogen, Carlsbad, CA, USA) according to the manufacturer's protocol. First-strand cDNA was synthesized using the High Capacity cDNA Reverse Transcription Kit (Applied Biosystems, Foster City, CA, USA). Quantitative real-time PCR was performed using SYBR Green PCR Master Mix (Applied Biosystems) with the 7900 Real-Time PCR System (Applied Biosystems). The PCR primers used to amplify GOLPH3 were as follows: 5′-GGGCGACTCCAAGGAAAC-3′ (forward) and 5′-CAGCCACGTAATCCAGATGAT-3′ (reverse) and β-actin: 5′-CATGTACGTTGCTATCCAGGC-3′ (forward) and 5′-CTCCTTAATGTCACGCACGA-3′ (reverse). β-actin was used as the reference gene. The Ct values of the samples were calculated, and the relative levels of GOLPH3 mRNA were analyzed by the 2^−△△^Ct method.

### Gene silencing by siRNA and shRNA

siRNA targeting GOLPH3 and negative control siRNA were purchased from Shanghai Genepharma Co. Ltd. (Shanghai, China). The siRNA sequences for GOLPH3 were 5′-GUUA AGAAAUGUACGGGAATT-3′ (siRNA#1) and 5′-GGUGAGACAUGGAAUCCAUTT-3′ (siRNA#2). Cells were transfected with either GOLPH3 or negative control siRNA using Lipofectamine^®^ 3000 Reagent (Invitrogen) according to the manufacturer's instructions. Following transfection, the mRNA and protein levels were assessed 48 hours later.

To silencing GOLPH3 again, GOLPH3-targeting siRNA#1 sequences were cloned into the pSuper-retro-puro vector to generate pSuper-retro-GOLPH3-RNAi. Retroviral production and infection were conducted as previously described^16^. Stable T24 bladder cancer cell lines expressing GOLPH3 (Lv-shRNA-GOLPH3 with GFP) and negative control (Lv-shRNA-NC with GFP) short hairpin RNAs were selected for 10 days with 0.5 mg/ml puromycin.

### Cell viability

Cell viability was measured with the CCK8 assay (cell counting kit-8, Vazyme, Piscataway, NJ, USA). Transfected (transfected for 24 hours with either GOLPH3 or negative control siRNA) and stable T24 cell lines expressing GOLPH3 or negative control short hairpin RNAs were prepared in 96-well cell culture plates at a cellular density of 2 × 10^3^ cells/well at 37°C for 24, 48 and 72 hours. The cell monolayer was washed three times with phosphate-buffered saline (PBS), and then CCK8 solution diluted 1:10 in RPMI 1640 was added to the cells and incubated for 2 h at 37°C. The results were measured with a microplate reader at 450 nm and expressed as a percentage of control values (obtained for cells treated with vehicle).

### Anchorage-independent growth ability assay

Five hundred transfected cells (transfected for 24 hours with siRNA) were trypsinized and resuspended in 2 ml complete medium plus 0.3% agar (Sigma-Aldrich, Louis, MO, USA). The agar cell mixture was plated on top of a bottom layer containing a 1% agar complete medium mixture. For approximately 10 days, viable colonies that were larger than 0.05 mm were counted.

### Flow cytometry

Cell cycle distribution was analyzed with a FACScan flow cytometer (BD Biosciences). At 48 h after transfection of siRNA (or stable T24 cell lines expressing GOLPH3 or negative control short hairpin RNAs), cells were harvested, washed three times with cold PBS, and then fixed in 70% ethanol in PBS at −20°C overnight. After fixation, cells were washed with cold PBS and stained with 1 ml of propidium iodide (PI) staining buffer, which contained 200 mg/ml RNase A (Sigma, R6513) and 50 μg/ml PI (Sigma, P4170), at room temperature for 30 min in the dark. The results were analyzed with CellQuest software.

### Scratch migration assay

Cells were seeded in 12-well plates and transfected with negative control or GOLPH3 siRNA. At 24 h after transfection, cells were scratched using the tip of a sterile 200 ml pipette in each well. The plates were washed twice with PBS to remove detached cells and then incubated at 37°C in 5% CO_2_. Wound closure was observed and measured after 24 h.

### Matrigel invasion assay

Cell invasion assays were performed using a 24-well transwell chamber with a pore size of 8 μm (Costar, New York, NY, USA). The inserts were coated with 50 μL Matrigel (dilution at 1 : 2; BD Bioscience, Franklin Lakes, NJ, USA). Cells were trypsinized after transfection with control or GOLPH3 siRNA for 24 h and transferred to the upper Matrigel chamber in 100 μL serum-free medium containing 1 × 10^5^ cells, and they were incubated for 24 h. The lower chamber was filled with medium containing 10% FBS as a chemoattractant. After incubation, the non-invading cells on the upper membrane surface were removed with a cotton tip, and the cells that passed through the filter were stained using crystal violet. The number of invading cells was counted in five randomly selected high power fields under a microscope.

### Xenograft tumor model

Four-week old male BALB/c nude mice (Experimental Animal Center of Nanjing Medical University, Nanjing, China) were housed in a specific pathogen free (SPF) environment at the Animal Laboratory Center, Drum Tower Hospital, Medical School of Nanjing University. Cells (5.0 × 10^6^ cell) were suspended in 100 ml PBS and subcutaneously injected in the right flank region of nude mice. Mice injected with stable T24 cell lines expressing GOLPH3 and negative control short hairpin RNAs nude mice with tumor burden were divided into two groups: Lv-shRNA-GOLPH3 and Lv-shRNA-NC groups (*n* = 6 for each group). Furthermore, we monitored the tumor volume using the IVIS Lumina II system (Caliper Life Sciences, Hopkinton, MA, USA) at the end point. After 10 days, the nude mice injected with T24 cells with a tumor burden were randomly divided into three groups (*n* = 4 for each group) to receive an intratumoral injection of PBS (15 μL), negative control siRNA or GOLPH3 siRNA complexes every 5 days. Each complex contained 10 mg siRNA and 7.5 ml Lipofectamine^®^ 3000 reagent (Invitrogen) in PBS (Total volume 15 μL). For each group, the tumor volumes were measured three times a week with a caliper and calculated according to the formula: length × width^2^/2. All mice were humanely killed after six treatments (ten days after the last time of treatment), and the resected tumors were weighed. All experimental manipulations were undertaken in accordance with the National Institutes of Health Guide for the Care and Use of Laboratory Animals with the approval of the Scientific Investigation Board of Drum Tower Hospital, Medical School of Nanjing University.

### Statistical analysis

All statistical analyses were conducted using SPSS version 17.0 statistical software (SPSS Inc., Chicago, IL, USA). Comparisons between groups for statistical significance were performed with a 2-tailed paired Student's *t* test. The relationship between GOLPH3 expression and clinicopathologic characteristics was analyzed by the χ^2^ test. Survival curves were plotted using the Kaplan-Meier method and compared using the log-rank test. Survival data were evaluated by univariate and multivariate cox regression analyses. Values are the mean ± SD of 3 independent experiments. * *P* < 0.05, ** *P* < 0.01, ***P < 0.001, relative to control.
